# (1*S*,3*S*,8*R*,9*S*,11*R*)-10,10-Di­bromo-3,7,7,11-tetra­methyl­tetra­cyclo­[6.5.0.0^1,3^.0^9,11^]trideca­ne

**DOI:** 10.1107/S160053681301903X

**Published:** 2013-07-13

**Authors:** Ahmed Benharref, Jamal El karroumi, Lahcen El Ammari, Mohamed Saadi, Moha Berraho

**Affiliations:** a Laboratoire de Chimie des Substances Naturelles, "Unité Associé au CNRST (URAC16)", Faculté des Sciences Semlalia, BP 2390 Bd My Abdellah, 40000 Marrakech, Morocco; b Laboratoire de Chimie du Solide Appliquée, Faculté des Sciences, Université MohammedV-Agdal, Avenue Ibn Battouta, BP 1014, Rabat, Morocco

## Abstract

The title compound, C_17_H_26_Br_2_, was synthesized from β-himachalene (3,5,5,9-tetra­methyl-2,4a,5,6,7,8-hexa­hydro-1*H*-benzo­cyclo­heptene), which was isolated from the essential oil of the Atlas cedar (*Cedrus Atlantica*). The asymmetric unit contains two independent mol­ecules with similar conformations. Each mol­ecule is built up from fused six- and seven-membered rings and two appended three-membered rings. In both mol­ecules, the six-membered ring has a screw boat conformation, whereas the seven-membered ring displays a boat conformation. No specific inter­molecular inter­actions were discerned in the crystal packing.

## Related literature

For backgroud to Moroccan floral heritage, see: Daoubi *et al.* (2004 ▶); Benharref *et al.* (2013 ▶); Oukhrib *et al.* (2013 ▶). For conformational analysis, see: Cremer & Pople (1975 ▶).
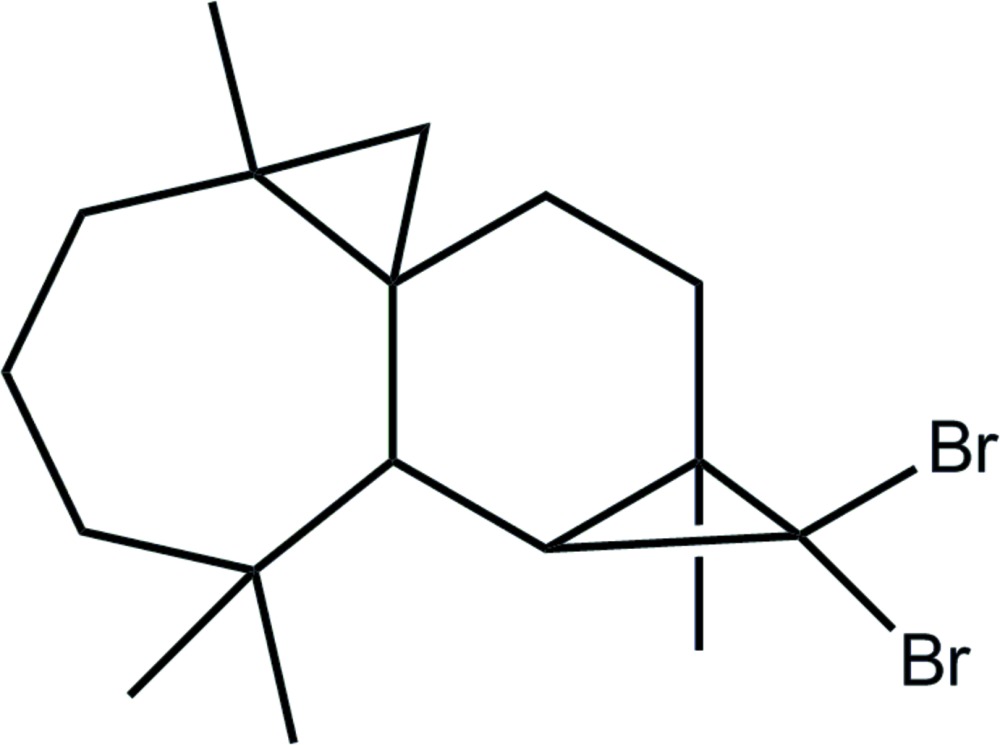


## Experimental

### 

#### Crystal data

C_17_H_26_Br_2_
*M* *_r_* = 390.20
Monoclinic, 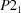

*a* = 6.585 (7) Å
*b* = 29.05 (3) Å
*c* = 9.385 (9) Å
β = 110.29 (2)°
*V* = 1684 (3) Å^3^
*Z* = 4
Mo *K*α radiation
μ = 4.80 mm^−1^
*T* = 296 K
0.20 × 0.15 × 0.12 mm

#### Data collection

Bruker APEXII CCD diffractometer
Absorption correction: multi-scan (*SADABS*; Bruker, 2009 ▶) *T*_min_ = 0.423, *T*_max_ = 0.617
11862 measured reflections
5595 independent reflections
4255 reflections with *I* > 2σ(*I*)
*R*_int_ = 0.041

#### Refinement

*R*[*F* ^2^ > 2σ(*F* ^2^)] = 0.042
*wR*(*F* ^2^) = 0.095
*S* = 1.10
5595 reflections
352 parameters
1 restraint
H-atom parameters constrained
Δρ_max_ = 0.57 e Å^−3^
Δρ_min_ = −0.34 e Å^−3^
Absolute structure: Flack & Bernardinelli (2000 ▶), 614 Friedel pairs
Absolute structure parameter: 0.019 (12)

### 

Data collection: *APEX2* (Bruker, 2009 ▶); cell refinement: *SAINT* (Bruker, 2009 ▶); data reduction: *SAINT*; program(s) used to solve structure: *SHELXS97* (Sheldrick, 2008 ▶); program(s) used to refine structure: *SHELXL97* (Sheldrick, 2008 ▶); molecular graphics: *ORTEP-3 for Windows* (Farrugia, 2012 ▶); software used to prepare material for publication: *WinGX* (Farrugia, 2012 ▶).

## Supplementary Material

Crystal structure: contains datablock(s) I, global. DOI: 10.1107/S160053681301903X/tk5238sup1.cif

Structure factors: contains datablock(s) I. DOI: 10.1107/S160053681301903X/tk5238Isup2.hkl

Supplementary material file. DOI: 10.1107/S160053681301903X/tk5238Isup3.cml

Additional supplementary materials:  crystallographic information; 3D view; checkCIF report

## Figures and Tables

**Fig. 1.**
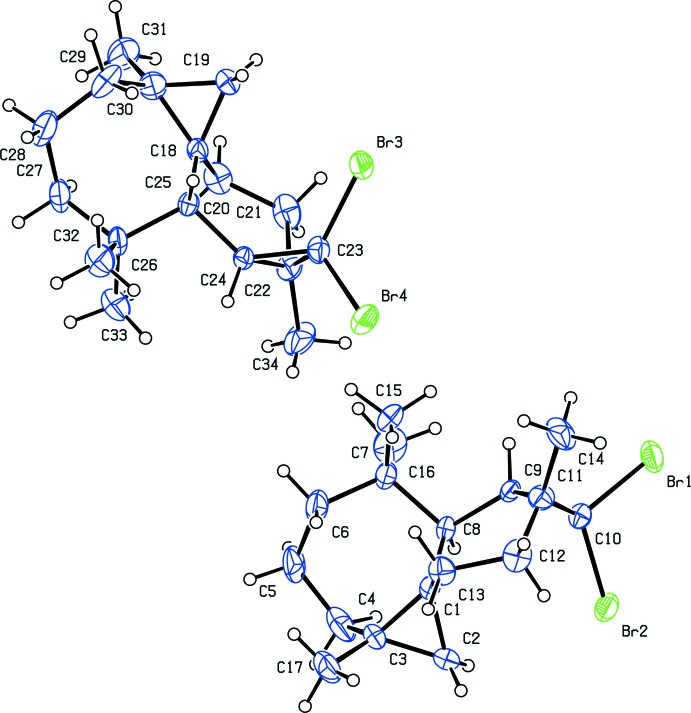
Molecular structure of the title compound with the atom-labelling scheme. Displacement ellipsoids are drawn at the 30% probability. level.

## supplementary crystallographic information

### Comment

As part of the development of our Moroccan floral heritage, we continue to study the reactivity of β-himachalene, constituting principal (50%) of the essential oil of the Atlas cedar (Cedrus Atlantica) (Oukhrib *et al.*, 2013; Benharref *et al.*, 2013), in order to prepare new products having biological properties (Daoubi *et al.*, 2004). In this work we present the crystal structure of the title compound, (1*S*,3S,8*R*,9S,11*R*)-10,10-dibromo-3,7,7,11-tetramethyl- tetracyclo-[6.5.0.01,3.09,11]tridecane.

Each molecule contains a fused six-and seven-membered rings, which is fused to two three-membered rings as shown in Fig. 1. The six-membered ring has a screw boat conformation, as indicated by the total puckering amplitude QT = 0.492 (6) Å and spherical polar angle θ = 139.6 (7)° with φ = 130.8 (1)°, whereas the seven-membered ring display a boat conformation with QT = 1.1449 (8) Å, θ = 88.49 (5)°, φ2 = -49.77 (5)° and φ3 = -127.91 (13)° (Cremer & Pople, 1975). Owing to the presence of Br atoms, the absolute configuration could be fully confirmed, by refining the Flack parameter (Flack & Bernardinelli (2000)) as C1(S), C3(S), C8(*R*),C9(S)and C11(*R*).

### Experimental

A solution containing 2 g (9 mmol) of (1*S*,3S,8*R*,9S,11*R*)- 3,7,7,11-tetramethyltri-*cyclo*-[6.5.0.0^1^,^3^]tridec-9-ene) (Benharref *et al.*, 2013) and 1 ml (10 mmol) of CHBr_3_ in 40 ml of dichloromethane was added drop-wise at 273 K over 30 min to 1 g of pulverized sodium hydroxide and 40 mg of *N*-benzyltriethylammonium chloride placed in a 100 ml three-necked flask. After stirring at room temperature for 2 h, the mixture was filtered on celite and impregnated with silver nitrate (10%) with a mixture of hexane-ethyl acetate (95:5 *v*/*v*) used as eluent. The two diastereoisomers (1*S*,3S,8*R*,9S,11*R*)-10,10-dibromo-3,7,7,11- tetramethyltetracyclo- [6.5.0.0^1^,^3^.0^9^,^11^]tridecane (*X*) and (1*S*,3S,8*R*,9*R*,11*S*)-10,10-dibromo-3,7,7,11- tetramethyltetracyclo-[6.5.0.0^1^,^3^.0^9^,^11^]tridecane (Y), were obtained by this procedure in a 85:15 ratio and a combined yield of 86% (3 g; 7.7 mmol). The title compound (isomer *X*) was recrystallized from n-heptane.

### Refinement

All H atoms were fixed geometrically and treated as riding with C—H = 0.96 Å (methyl), 0.97 Å (methylene) and 0.98 Å (methine) with *U*_iso_(H) = 1.2*U*_eq_(methylene, methine) or *U*_iso_(H) = 1.5*U*_eq_(methyl).

### Crystal data

**Table:**

C_17_H_26_Br_2_	*F*(000) = 792
*M**_r_* = 390.20	*D*_x_ = 1.539 Mg m^−^^3^
Monoclinic, *P*2_1_	Mo *K*α radiation, λ = 0.71073 Å
Hall symbol: P 2yb	Cell parameters from 5595 reflections
*a* = 6.585 (7) Å	θ = 2.3–26.4°
*b* = 29.05 (3) Å	µ = 4.80 mm^−^^1^
*c* = 9.385 (9) Å	*T* = 296 K
β = 110.29 (2)°	Box, colourless
*V* = 1684 (3) Å^3^	0.20 × 0.15 × 0.12 mm
*Z* = 4	

### Data collection

**Table:**

Bruker APEXII CCD diffractometer	5595 independent reflections
Radiation source: fine-focus sealed tube	4255 reflections with *I* > 2σ(*I*)
Graphite monochromator	*R*_int_ = 0.041
ω and φ scans	θ_max_ = 26.4°, θ_min_ = 2.3°
Absorption correction: multi-scan (*SADABS*; Bruker, 2009)	*h* = −8→8
*T*_min_ = 0.423, *T*_max_ = 0.617	*k* = −36→28
11862 measured reflections	*l* = −11→11

### Refinement

**Table:**

Refinement on *F*^2^	Secondary atom site location: difference Fourier map
Least-squares matrix: full	Hydrogen site location: inferred from neighbouring sites
*R*[*F*^2^ > 2σ(*F*^2^)] = 0.042	H-atom parameters constrained
*wR*(*F*^2^) = 0.095	*w* = 1/[σ^2^(*F*_o_^2^) + (0.0325*P*)^2^ + 0.0388*P*] where *P* = (*F*_o_^2^ + 2*F*_c_^2^)/3
*S* = 1.10	(Δ/σ)_max_ < 0.001
5595 reflections	Δρ_max_ = 0.57 e Å^−^^3^
352 parameters	Δρ_min_ = −0.34 e Å^−^^3^
1 restraint	Absolute structure: Flack & Bernardinelli (2000), 614 Friedel pairs
Primary atom site location: structure-invariant direct methods	Flack parameter: 0.019 (12)

### Special details

**Table:**

Geometry. All s.u.'s (except the s.u. in the dihedral angle between two l.s. planes) are estimated using the full covariance matrix. The cell s.u.'s are taken into account individually in the estimation of s.u.'s in distances, angles and torsion angles; correlations between s.u.'s in cell parameters are only used when they are defined by crystal symmetry. An approximate (isotropic) treatment of cell s.u.'s is used for estimating s.u.'s involving l.s. planes.
Refinement. Refinement of *F*^2^ against all reflections. The weighted *R*-factor *wR* and goodness of fit *S* are based on *F*^2^, conventional *R*-factors *R* are based on *F*, with *F* set to zero for negative *F*^2^. The threshold expression of *F*^2^ > 2σ(*F*^2^) is used only for calculating *R*-factors(gt) *etc*. and is not relevant to the choice of reflections for refinement. *R*-factors based on *F*^2^ are statistically about twice as large as those based on *F*, and *R*- factors based on all data will be even larger.

### Fractional atomic coordinates and isotropic or equivalent isotropic displacement parameters (Å^2^)

**Table:**

	*x*	*y*	*z*	*U*_iso_*/*U*_eq_	
C1	0.4761 (9)	0.1497 (2)	−0.2261 (6)	0.0368 (14)	
C2	0.6203 (13)	0.1079 (3)	−0.1740 (8)	0.062 (2)	
H2A	0.7757	0.1128	−0.1346	0.074*	
H2B	0.5695	0.0832	−0.1253	0.074*	
C3	0.5052 (12)	0.1153 (2)	−0.3396 (7)	0.0525 (17)	
C4	0.6364 (13)	0.1306 (3)	−0.4324 (10)	0.074 (3)	
H4A	0.6741	0.1040	−0.4804	0.088*	
H4B	0.7699	0.1444	−0.3658	0.088*	
C5	0.5191 (13)	0.1650 (3)	−0.5532 (8)	0.067 (2)	
H5A	0.6255	0.1826	−0.5803	0.081*	
H5B	0.4315	0.1483	−0.6430	0.081*	
C6	0.3757 (13)	0.1980 (3)	−0.5084 (8)	0.061 (2)	
H6A	0.2586	0.1802	−0.4952	0.074*	
H6B	0.3113	0.2186	−0.5933	0.074*	
C7	0.4736 (10)	0.2272 (2)	−0.3674 (7)	0.0436 (16)	
C8	0.5756 (9)	0.1969 (2)	−0.2176 (6)	0.0302 (13)	
H8	0.7286	0.1923	−0.2040	0.036*	
C9	0.5691 (9)	0.2249 (2)	−0.0811 (6)	0.0323 (13)	
H9	0.5674	0.2581	−0.1007	0.039*	
C10	0.6687 (10)	0.2146 (2)	0.0815 (7)	0.0403 (15)	
C11	0.4226 (10)	0.2126 (2)	0.0055 (8)	0.0428 (16)	
C12	0.3200 (10)	0.1654 (3)	−0.0218 (8)	0.0513 (18)	
H12A	0.1867	0.1660	0.0003	0.062*	
H12B	0.4171	0.1434	0.0463	0.062*	
C13	0.2710 (9)	0.1499 (2)	−0.1868 (7)	0.0467 (17)	
H13A	0.2090	0.1192	−0.2005	0.056*	
H13B	0.1658	0.1705	−0.2548	0.056*	
C14	0.2810 (12)	0.2499 (3)	0.0397 (9)	0.070 (2)	
H14A	0.1414	0.2502	−0.0392	0.104*	
H14B	0.3488	0.2794	0.0442	0.104*	
H14C	0.2644	0.2435	0.1353	0.104*	
C15	0.2804 (12)	0.2568 (3)	−0.3617 (8)	0.070 (2)	
H15A	0.2147	0.2721	−0.4574	0.106*	
H15B	0.3314	0.2793	−0.2823	0.106*	
H15C	0.1756	0.2372	−0.3419	0.106*	
C16	0.6498 (15)	0.2587 (3)	−0.3829 (10)	0.085 (3)	
H16A	0.7577	0.2406	−0.4042	0.128*	
H16B	0.7154	0.2754	−0.2898	0.128*	
H16C	0.5871	0.2801	−0.4644	0.128*	
C17	0.3222 (14)	0.0823 (3)	−0.4235 (9)	0.075 (2)	
H17A	0.2141	0.0985	−0.5034	0.113*	
H17B	0.2586	0.0700	−0.3537	0.113*	
H17C	0.3792	0.0575	−0.4661	0.113*	
C18	0.9410 (9)	0.4778 (2)	0.0423 (6)	0.0364 (14)	
C19	0.8677 (11)	0.5225 (2)	0.0944 (8)	0.0507 (17)	
H19A	0.7507	0.5207	0.1347	0.061*	
H19B	0.9779	0.5452	0.1431	0.061*	
C30	1.1803 (10)	0.4724 (3)	0.0799 (8)	0.0533 (18)	
H30A	1.2428	0.5015	0.0664	0.064*	
H30B	1.2077	0.4501	0.0117	0.064*	
C29	1.2851 (10)	0.4563 (3)	0.2431 (8)	0.059 (2)	
H29A	1.4386	0.4516	0.2640	0.071*	
H29B	1.2698	0.4802	0.3107	0.071*	
C28	1.1879 (9)	0.4121 (3)	0.2764 (8)	0.0481 (17)	
C27	1.0195 (9)	0.4153 (2)	0.3516 (7)	0.0426 (15)	
C26	0.9492 (8)	0.4037 (2)	0.1862 (6)	0.0341 (13)	
H26	0.9168	0.3709	0.1676	0.041*	
C25	0.8217 (8)	0.4331 (2)	0.0498 (6)	0.0325 (13)	
H25	0.6855	0.4416	0.0633	0.039*	
C24	0.7622 (10)	0.4018 (2)	−0.0963 (7)	0.0425 (16)	
C23	0.7287 (12)	0.4304 (3)	−0.2444 (8)	0.059 (2)	
H23A	0.8652	0.4452	−0.2338	0.071*	
H23B	0.6980	0.4089	−0.3283	0.071*	
C22	0.5528 (13)	0.4673 (3)	−0.2892 (8)	0.073 (2)	
H22A	0.4126	0.4526	−0.3141	0.088*	
H22B	0.5562	0.4829	−0.3797	0.088*	
C21	0.5771 (12)	0.5033 (3)	−0.1642 (9)	0.067 (2)	
H21A	0.5023	0.5312	−0.2103	0.080*	
H21B	0.5085	0.4918	−0.0951	0.080*	
C20	0.8121 (12)	0.5148 (3)	−0.0742 (8)	0.0542 (18)	
C31	0.9226 (16)	0.5428 (3)	−0.1623 (9)	0.083 (3)	
H31A	0.8482	0.5716	−0.1921	0.124*	
H31B	0.9195	0.5261	−0.2512	0.124*	
H31C	1.0701	0.5485	−0.0995	0.124*	
C32	0.5532 (11)	0.3758 (3)	−0.1142 (9)	0.062 (2)	
H32A	0.5154	0.3567	−0.2031	0.093*	
H32B	0.4388	0.3974	−0.1245	0.093*	
H32C	0.5739	0.3569	−0.0263	0.093*	
C33	0.9350 (11)	0.3668 (3)	−0.0930 (8)	0.065 (2)	
H33A	0.9501	0.3450	−0.0133	0.097*	
H33B	1.0704	0.3824	−0.0748	0.097*	
H33C	0.8939	0.3511	−0.1888	0.097*	
C34	1.3466 (12)	0.3709 (4)	0.3125 (9)	0.077 (3)	
H34A	1.4120	0.3687	0.2359	0.115*	
H34B	1.2691	0.3431	0.3142	0.115*	
H34C	1.4571	0.3755	0.4099	0.115*	
Br1	0.79397 (12)	0.26543 (3)	0.21731 (8)	0.0588 (2)	
Br2	0.83916 (11)	0.16088 (2)	0.16108 (7)	0.0514 (2)	
Br3	0.94881 (11)	0.47197 (3)	0.42705 (8)	0.0555 (2)	
Br4	0.99939 (14)	0.36640 (3)	0.48505 (9)	0.0678 (2)	

### Atomic displacement parameters (Å^2^)

**Table:**

	*U*^11^	*U*^22^	*U*^33^	*U*^12^	*U*^13^	*U*^23^
C1	0.039 (3)	0.041 (4)	0.028 (3)	0.004 (3)	0.008 (3)	0.002 (3)
C2	0.093 (6)	0.036 (4)	0.048 (5)	0.018 (4)	0.014 (4)	−0.005 (3)
C3	0.079 (5)	0.039 (4)	0.036 (4)	0.001 (4)	0.015 (4)	−0.009 (3)
C4	0.078 (6)	0.078 (7)	0.067 (6)	0.000 (5)	0.026 (5)	−0.036 (5)
C5	0.095 (6)	0.083 (6)	0.031 (4)	−0.018 (5)	0.032 (4)	−0.008 (4)
C6	0.079 (5)	0.069 (6)	0.031 (4)	−0.011 (4)	0.013 (4)	0.004 (4)
C7	0.048 (4)	0.048 (4)	0.030 (4)	−0.006 (3)	0.006 (3)	0.004 (3)
C8	0.025 (3)	0.042 (4)	0.023 (3)	−0.003 (2)	0.008 (3)	0.002 (3)
C9	0.035 (3)	0.030 (3)	0.027 (3)	−0.005 (2)	0.003 (3)	0.005 (3)
C10	0.045 (4)	0.043 (4)	0.033 (4)	0.001 (3)	0.014 (3)	0.004 (3)
C11	0.041 (4)	0.046 (4)	0.045 (4)	0.009 (3)	0.020 (3)	0.001 (3)
C12	0.038 (3)	0.070 (5)	0.053 (4)	−0.012 (3)	0.025 (3)	0.005 (4)
C13	0.040 (3)	0.049 (4)	0.051 (4)	−0.013 (3)	0.015 (3)	−0.004 (3)
C14	0.069 (5)	0.077 (6)	0.066 (6)	0.015 (4)	0.027 (4)	−0.026 (4)
C15	0.088 (6)	0.062 (6)	0.043 (5)	0.026 (4)	−0.002 (4)	0.011 (4)
C16	0.123 (7)	0.069 (6)	0.069 (6)	−0.039 (5)	0.039 (6)	0.014 (5)
C17	0.110 (7)	0.052 (5)	0.058 (5)	−0.022 (5)	0.022 (5)	−0.024 (4)
C18	0.040 (3)	0.034 (4)	0.031 (3)	−0.010 (3)	0.007 (3)	−0.004 (3)
C19	0.064 (4)	0.041 (4)	0.041 (4)	−0.003 (3)	0.010 (3)	−0.007 (3)
C30	0.054 (4)	0.058 (4)	0.055 (5)	−0.020 (4)	0.029 (4)	−0.007 (4)
C29	0.031 (4)	0.084 (6)	0.058 (5)	−0.010 (3)	0.011 (4)	−0.017 (4)
C28	0.032 (3)	0.058 (5)	0.045 (4)	0.011 (3)	0.002 (3)	−0.001 (3)
C27	0.038 (3)	0.052 (4)	0.034 (4)	0.002 (3)	0.008 (3)	−0.004 (3)
C26	0.031 (3)	0.037 (4)	0.029 (4)	0.002 (2)	0.004 (3)	−0.002 (3)
C25	0.025 (3)	0.040 (4)	0.030 (4)	−0.001 (2)	0.006 (3)	0.001 (3)
C24	0.047 (4)	0.050 (4)	0.026 (4)	−0.010 (3)	0.006 (3)	−0.007 (3)
C23	0.071 (5)	0.070 (6)	0.030 (4)	−0.013 (4)	0.010 (4)	−0.010 (4)
C22	0.077 (6)	0.085 (7)	0.041 (5)	−0.006 (5)	0.000 (4)	0.016 (5)
C21	0.058 (5)	0.083 (6)	0.051 (5)	0.014 (4)	0.009 (4)	0.032 (5)
C20	0.068 (5)	0.045 (5)	0.051 (5)	−0.011 (4)	0.023 (4)	0.003 (3)
C31	0.123 (8)	0.061 (6)	0.063 (6)	−0.013 (5)	0.030 (6)	0.015 (5)
C32	0.048 (4)	0.063 (6)	0.064 (5)	−0.023 (4)	0.005 (4)	−0.008 (4)
C33	0.072 (5)	0.065 (5)	0.061 (5)	−0.002 (4)	0.027 (4)	−0.020 (4)
C34	0.062 (5)	0.104 (7)	0.061 (5)	0.048 (5)	0.015 (4)	0.018 (5)
Br1	0.0718 (5)	0.0608 (5)	0.0380 (4)	−0.0117 (4)	0.0117 (4)	−0.0143 (4)
Br2	0.0515 (4)	0.0618 (5)	0.0357 (4)	0.0123 (3)	0.0083 (3)	0.0071 (3)
Br3	0.0632 (4)	0.0594 (5)	0.0394 (4)	0.0123 (4)	0.0121 (3)	−0.0079 (4)
Br4	0.0893 (5)	0.0676 (6)	0.0441 (5)	0.0164 (4)	0.0202 (4)	0.0179 (4)

### Geometric parameters (Å, º)

**Table:**

C1—C8	1.510 (8)	C18—C30	1.499 (9)
C1—C2	1.516 (9)	C18—C19	1.522 (9)
C1—C13	1.519 (8)	C18—C25	1.533 (8)
C1—C3	1.520 (8)	C18—C20	1.559 (10)
C2—C3	1.490 (10)	C19—C20	1.512 (10)
C2—H2A	0.9700	C19—H19A	0.9700
C2—H2B	0.9700	C19—H19B	0.9700
C3—C4	1.492 (10)	C30—C29	1.519 (10)
C3—C17	1.528 (10)	C30—H30A	0.9700
C4—C5	1.507 (12)	C30—H30B	0.9700
C4—H4A	0.9700	C29—C28	1.515 (10)
C4—H4B	0.9700	C29—H29A	0.9700
C5—C6	1.504 (11)	C29—H29B	0.9700
C5—H5A	0.9700	C28—C27	1.510 (8)
C5—H5B	0.9700	C28—C26	1.524 (8)
C6—C7	1.516 (10)	C28—C34	1.545 (10)
C6—H6A	0.9700	C27—C26	1.496 (8)
C6—H6B	0.9700	C27—Br3	1.913 (7)
C7—C16	1.524 (9)	C27—Br4	1.927 (7)
C7—C15	1.551 (10)	C26—C25	1.524 (8)
C7—C8	1.596 (9)	C26—H26	0.9800
C8—C9	1.531 (8)	C25—C24	1.578 (8)
C8—H8	0.9800	C25—H25	0.9800
C9—C10	1.468 (9)	C24—C33	1.517 (10)
C9—C11	1.505 (8)	C24—C32	1.527 (9)
C9—H9	0.9800	C24—C23	1.568 (10)
C10—C11	1.529 (9)	C23—C22	1.528 (11)
C10—Br2	1.917 (6)	C23—H23A	0.9700
C10—Br1	1.938 (7)	C23—H23B	0.9700
C11—C12	1.512 (10)	C22—C21	1.538 (12)
C11—C14	1.534 (9)	C22—H22A	0.9700
C12—C13	1.536 (9)	C22—H22B	0.9700
C12—H12A	0.9700	C21—C20	1.521 (11)
C12—H12B	0.9700	C21—H21A	0.9700
C13—H13A	0.9700	C21—H21B	0.9700
C13—H13B	0.9700	C20—C31	1.513 (10)
C14—H14A	0.9600	C31—H31A	0.9600
C14—H14B	0.9600	C31—H31B	0.9600
C14—H14C	0.9600	C31—H31C	0.9600
C15—H15A	0.9600	C32—H32A	0.9600
C15—H15B	0.9600	C32—H32B	0.9600
C15—H15C	0.9600	C32—H32C	0.9600
C16—H16A	0.9600	C33—H33A	0.9600
C16—H16B	0.9600	C33—H33B	0.9600
C16—H16C	0.9600	C33—H33C	0.9600
C17—H17A	0.9600	C34—H34A	0.9600
C17—H17B	0.9600	C34—H34B	0.9600
C17—H17C	0.9600	C34—H34C	0.9600
			
C8—C1—C2	120.0 (5)	C30—C18—C19	116.0 (5)
C8—C1—C13	113.0 (5)	C30—C18—C25	114.4 (6)
C2—C1—C13	115.8 (6)	C19—C18—C25	118.9 (5)
C8—C1—C3	118.5 (5)	C30—C18—C20	120.7 (5)
C2—C1—C3	58.8 (4)	C19—C18—C20	58.8 (4)
C13—C1—C3	120.7 (5)	C25—C18—C20	117.1 (5)
C3—C2—C1	60.8 (4)	C20—C19—C18	61.8 (4)
C3—C2—H2A	117.7	C20—C19—H19A	117.6
C1—C2—H2A	117.7	C18—C19—H19A	117.6
C3—C2—H2B	117.7	C20—C19—H19B	117.6
C1—C2—H2B	117.7	C18—C19—H19B	117.6
H2A—C2—H2B	114.8	H19A—C19—H19B	114.7
C2—C3—C4	117.6 (7)	C18—C30—C29	110.1 (5)
C2—C3—C1	60.5 (4)	C18—C30—H30A	109.6
C4—C3—C1	116.8 (6)	C29—C30—H30A	109.6
C2—C3—C17	118.2 (7)	C18—C30—H30B	109.6
C4—C3—C17	113.5 (6)	C29—C30—H30B	109.6
C1—C3—C17	120.4 (6)	H30A—C30—H30B	108.2
C3—C4—C5	112.8 (7)	C28—C29—C30	113.1 (6)
C3—C4—H4A	109.0	C28—C29—H29A	109.0
C5—C4—H4A	109.0	C30—C29—H29A	109.0
C3—C4—H4B	109.0	C28—C29—H29B	109.0
C5—C4—H4B	109.0	C30—C29—H29B	109.0
H4A—C4—H4B	107.8	H29A—C29—H29B	107.8
C6—C5—C4	114.8 (5)	C27—C28—C29	118.4 (6)
C6—C5—H5A	108.6	C27—C28—C26	59.1 (4)
C4—C5—H5A	108.6	C29—C28—C26	116.6 (6)
C6—C5—H5B	108.6	C27—C28—C34	119.7 (6)
C4—C5—H5B	108.6	C29—C28—C34	113.5 (6)
H5A—C5—H5B	107.6	C26—C28—C34	119.2 (7)
C5—C6—C7	118.9 (7)	C26—C27—C28	60.9 (4)
C5—C6—H6A	107.6	C26—C27—Br3	123.3 (5)
C7—C6—H6A	107.6	C28—C27—Br3	122.6 (5)
C5—C6—H6B	107.6	C26—C27—Br4	116.3 (5)
C7—C6—H6B	107.6	C28—C27—Br4	119.0 (5)
H6A—C6—H6B	107.0	Br3—C27—Br4	108.3 (3)
C6—C7—C16	111.2 (6)	C27—C26—C25	128.9 (5)
C6—C7—C15	103.6 (6)	C27—C26—C28	60.0 (4)
C16—C7—C15	109.4 (7)	C25—C26—C28	122.7 (5)
C6—C7—C8	112.4 (6)	C27—C26—H26	111.8
C16—C7—C8	108.3 (6)	C25—C26—H26	111.8
C15—C7—C8	112.0 (5)	C28—C26—H26	111.8
C1—C8—C9	113.0 (4)	C26—C25—C18	111.8 (5)
C1—C8—C7	114.3 (5)	C26—C25—C24	107.8 (5)
C9—C8—C7	108.6 (5)	C18—C25—C24	115.2 (4)
C1—C8—H8	106.8	C26—C25—H25	107.2
C9—C8—H8	106.8	C18—C25—H25	107.2
C7—C8—H8	106.8	C24—C25—H25	107.2
C10—C9—C11	61.9 (4)	C33—C24—C32	108.2 (6)
C10—C9—C8	129.0 (5)	C33—C24—C23	104.3 (5)
C11—C9—C8	122.3 (5)	C32—C24—C23	108.7 (6)
C10—C9—H9	111.6	C33—C24—C25	114.2 (5)
C11—C9—H9	111.6	C32—C24—C25	108.8 (5)
C8—C9—H9	111.6	C23—C24—C25	112.4 (6)
C9—C10—C11	60.2 (4)	C22—C23—C24	118.5 (6)
C9—C10—Br2	124.2 (4)	C22—C23—H23A	107.7
C11—C10—Br2	122.3 (4)	C24—C23—H23A	107.7
C9—C10—Br1	117.6 (4)	C22—C23—H23B	107.7
C11—C10—Br1	118.5 (4)	C24—C23—H23B	107.7
Br2—C10—Br1	107.8 (3)	H23A—C23—H23B	107.1
C9—C11—C12	117.2 (5)	C23—C22—C21	113.5 (6)
C9—C11—C10	57.9 (4)	C23—C22—H22A	108.9
C12—C11—C10	116.9 (5)	C21—C22—H22A	108.9
C9—C11—C14	119.7 (6)	C23—C22—H22B	108.9
C12—C11—C14	114.1 (5)	C21—C22—H22B	108.9
C10—C11—C14	119.9 (6)	H22A—C22—H22B	107.7
C11—C12—C13	111.3 (5)	C20—C21—C22	112.9 (6)
C11—C12—H12A	109.4	C20—C21—H21A	109.0
C13—C12—H12A	109.4	C22—C21—H21A	109.0
C11—C12—H12B	109.4	C20—C21—H21B	109.0
C13—C12—H12B	109.4	C22—C21—H21B	109.0
H12A—C12—H12B	108.0	H21A—C21—H21B	107.8
C1—C13—C12	110.6 (5)	C19—C20—C31	120.4 (7)
C1—C13—H13A	109.5	C19—C20—C21	116.3 (6)
C12—C13—H13A	109.5	C31—C20—C21	113.9 (7)
C1—C13—H13B	109.5	C19—C20—C18	59.4 (4)
C12—C13—H13B	109.5	C31—C20—C18	120.4 (6)
H13A—C13—H13B	108.1	C21—C20—C18	116.0 (6)
C11—C14—H14A	109.5	C20—C31—H31A	109.5
C11—C14—H14B	109.5	C20—C31—H31B	109.5
H14A—C14—H14B	109.5	H31A—C31—H31B	109.5
C11—C14—H14C	109.5	C20—C31—H31C	109.5
H14A—C14—H14C	109.5	H31A—C31—H31C	109.5
H14B—C14—H14C	109.5	H31B—C31—H31C	109.5
C7—C15—H15A	109.5	C24—C32—H32A	109.5
C7—C15—H15B	109.5	C24—C32—H32B	109.5
H15A—C15—H15B	109.5	H32A—C32—H32B	109.5
C7—C15—H15C	109.5	C24—C32—H32C	109.5
H15A—C15—H15C	109.5	H32A—C32—H32C	109.5
H15B—C15—H15C	109.5	H32B—C32—H32C	109.5
C7—C16—H16A	109.5	C24—C33—H33A	109.5
C7—C16—H16B	109.5	C24—C33—H33B	109.5
H16A—C16—H16B	109.5	H33A—C33—H33B	109.5
C7—C16—H16C	109.5	C24—C33—H33C	109.5
H16A—C16—H16C	109.5	H33A—C33—H33C	109.5
H16B—C16—H16C	109.5	H33B—C33—H33C	109.5
C3—C17—H17A	109.5	C28—C34—H34A	109.5
C3—C17—H17B	109.5	C28—C34—H34B	109.5
H17A—C17—H17B	109.5	H34A—C34—H34B	109.5
C3—C17—H17C	109.5	C28—C34—H34C	109.5
H17A—C17—H17C	109.5	H34A—C34—H34C	109.5
H17B—C17—H17C	109.5	H34B—C34—H34C	109.5
